# Myogenic Tone in Peripheral Resistance Arteries and Arterioles: The Pressure Is On!

**DOI:** 10.3389/fphys.2021.699517

**Published:** 2021-07-22

**Authors:** William F. Jackson

**Affiliations:** Department of Pharmacology and Toxicology, College of Osteopathic Medicine, Michigan State University, East Lansing, MI, United States

**Keywords:** myogenic tone, arterioles, resistance arteries, ion channels, G-proteins, mechanotransduction

## Abstract

Resistance arteries and downstream arterioles in the peripheral microcirculation contribute substantially to peripheral vascular resistance, control of blood pressure, the distribution of blood flow to and within tissues, capillary pressure, and microvascular fluid exchange. A hall-mark feature of these vessels is myogenic tone. This pressure-induced, steady-state level of vascular smooth muscle activity maintains arteriolar and resistance artery internal diameter at 50–80% of their maximum passive diameter providing these vessels with the ability to dilate, reducing vascular resistance, and increasing blood flow, or constrict to produce the opposite effect. Despite the central importance of resistance artery and arteriolar myogenic tone in cardiovascular physiology and pathophysiology, our understanding of signaling pathways underlying this key microvascular property remains incomplete. This brief review will present our current understanding of the multiple mechanisms that appear to underlie myogenic tone, including the roles played by G-protein-coupled receptors, a variety of ion channels, and several kinases that have been linked to pressure-induced, steady-state activity of vascular smooth muscle cells (VSMCs) in the wall of resistance arteries and arterioles. Emphasis will be placed on the portions of the signaling pathways underlying myogenic tone for which there is lack of consensus in the literature and areas where our understanding is clearly incomplete.

## Introduction

Myogenic tone is a hall-mark feature of resistance arteries and their downstream arterioles. This pressure-induced contractile activity of vascular smooth muscle contributes substantially to all functions of the resistance vasculature, including maintenance of peripheral vascular resistance, control of blood pressure, distribution of blood flow to and within tissues, and regulation of capillary pressure and microvascular fluid exchange. However, our understanding of the molecular mechanisms responsible for myogenic tone remains incomplete. This review will outline the multiple mechanisms that appear to underlie this key microvascular process, including important roles for G-protein-coupled receptors, multiple ion channels, and several protein kinases emphasizing the portions of the signaling pathways for which there is a lack of consensus.

## Setting the Stage

### What Are Resistance Arteries and Arterioles?

Resistance arteries are arterial vessels that feed blood flow to the microcirculation and contribute to peripheral vascular resistance (Zweifach and Lipowsky, 1984; Segal, 2000). These small arteries have internal, maximal diameters ranging from 500 to 100 μm and have two or more layers of vascular smooth muscle in their walls. Arterioles are downstream from resistance arteries, usually have internal, maximal diameters less than 100 μm, and importantly, have only a single layer of vascular smooth muscle wrapped circumferentially around the endothelial cell tube that forms the lumen of these microvessels. Another distinguishing characteristic of arterioles is that they are usually embedded within the parenchyma to which they supply blood flow. Arterioles form a branching network of vessels that ultimately provide blood flow to the capillary bed, with 3–5 levels of branching, dependent on the tissue/organ being perfused. The last arterial microvessels with vascular smooth muscle cells (VSMCs) in their walls are termed terminal arterioles which then branch into 1–20 capillaries. As with resistance arteries, arterioles contribute substantially to determination and control of vascular resistance and blood pressure (Renkin, 1984; Zweifach and Lipowsky, 1984; Pries and Secomb, 2011).

In skeletal muscle, for example, resistance arteries contribute about 30–40% of total skeletal muscle vascular resistance (Segal and Duling, 1986; Segal, 2000), with downstream arterioles contributing 50% and capillary and venules contributing the remainder of the hydraulic resistance (Fronek and Zweifach, 1975). Thus, for example, during skeletal muscle contraction, when blood flow can increase 100-fold (Saltin et al., 1998; Mortensen and Saltin, 2014), coordinated vasodilation of arterioles in the microcirculation and upstream resistance arteries is essential for attainment of these massive increases in blood flow (Segal and Duling, 1986; Segal, 2000).

### What Are the Myogenic Response and Myogenic Tone?

**Figure 1:**
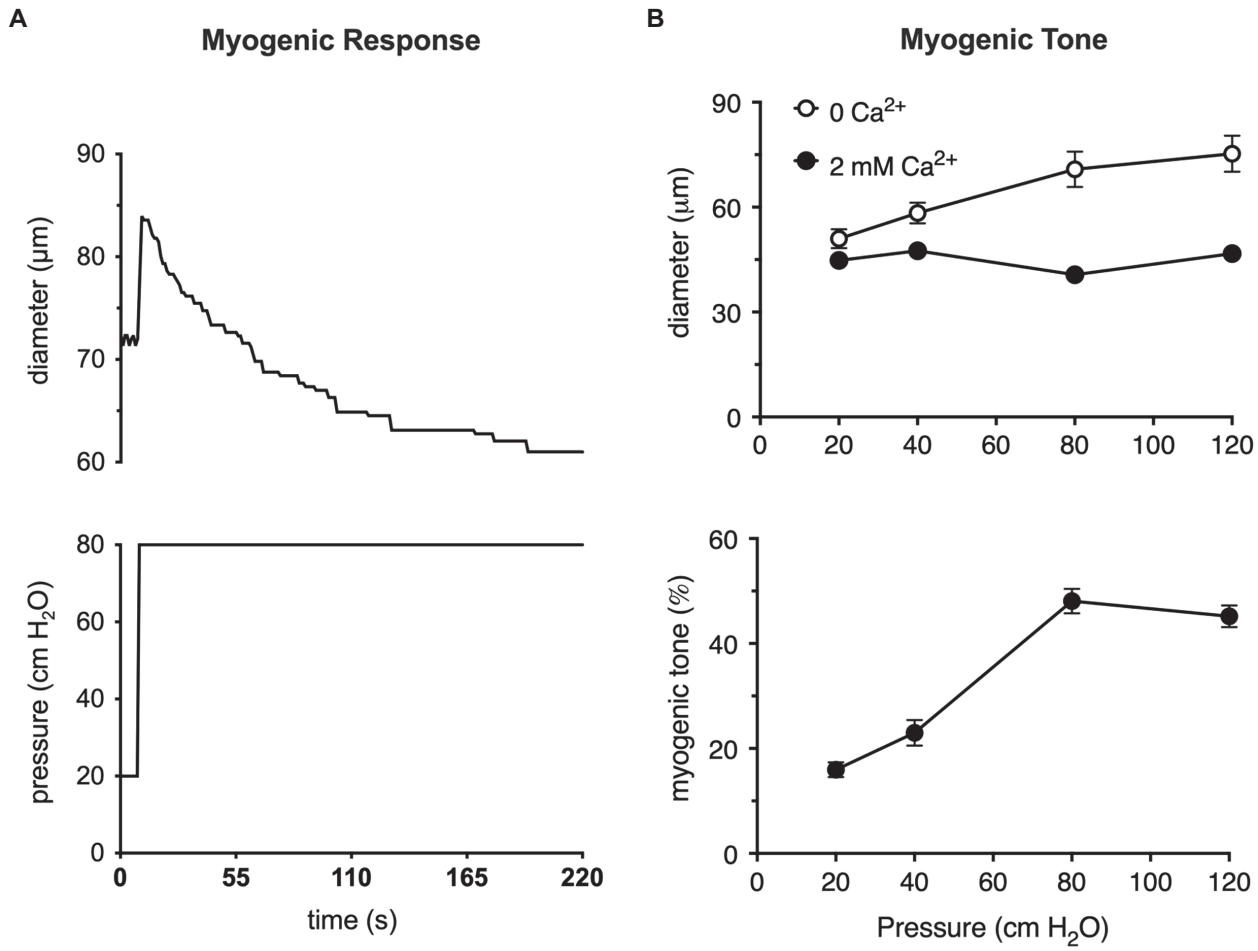
The myogenic response and myogenic tone in arterioles. **(A)** Myogenic response in a cannulated hamster cremaster arteriole, *ex vivo*, prepared as described (Westcott and Jackson, 2011). Shown in the upper panel is a digitized diameter record of the response of a second-order cremaster arteriole to a step-increase in luminal pressure from 20 to 80 cm H_2_O as depicted in the lower panel. At the onset of the pressure step, arteriolar diameter increases due to passive distention of the vessel. As the smooth muscle responds and begins to contract, diameter recovers to a new steady-state diameter that is slightly less than the diameter at 20 cm H_2_O. This behavior is the myogenic response. **(B)** Myogenic tone in cannulated hamster cremaster arterioles. Top panel shows the steady-state diameters of arterioles at different pressures in the absence of extracellular Ca^2+^ (passive) and presence of 2 mM Ca^2+^ (active). Bottom panel shows the % myogenic tone at the given pressures computed from the data in the top panel as myogenic tone = (passive diameter–active diameter)/passive diameter × 100%. At pressures greater than 20 cm H_2_O, arterioles develop significant myogenic tone (i.e., steady-state pressure-induced constriction). Data in **(A)** are replotted from Jackson (2012), with permission. Data in **(B)** are replotted from Westcott and Jackson, (2011) and Jackson, (2012), with permission.

A step-wise increase in the blood pressure within a resistance artery or arteriole leads to a rapid, pressure-induced increase in vessel diameter as shown in Figure 1A. If the pressure is maintained, the smooth muscle in the wall of the vessel will respond, contracting and returning the internal diameter of the vessel to or below its initial diameter (Figure 1A). This is the classic myogenic response (Johnson, 1980; Davis and Hill, 1999; Hill et al., 2001; Davis et al., 2011) that was originally described by Bayliss over a century ago (Bayliss, 1902). The steady-state level of contractile activity of the vascular smooth muscle in a pressurized blood vessel is myogenic tone (Figure 1B). It should be noted that myogenic tone not only encompasses the steady-state activity of the smooth muscle contractile machinery (actin-myosin cross bridge cycling), but also remodeling of the actin cytoskeleton (Gunst and Zhang, 2008) and alterations in interactions of smooth muscle cells with the extracellular matrix that accompanies maintained vasoconstriction (Martinez-Lemus et al., 2004) or vasodilation (Clifford et al., 2018). An important point is that the time scale for these latter events (remodeling of the actin cytoskeleton and interactions and remodeling of the extracellular matrix) may occur on much longer time scales than simple Ca^2+^-dependent cross-bridge cycling during maintained levels of myogenic tone.

These two processes, the myogenic response and myogenic tone (Figure 1), participate in the blood flow autoregulation in organs, such as the brain (Tuma, 2011), heart (Laughlin et al., 1996; Zhang et al., 2011), kidney (Navar et al., 2011), eye (Riva and Schmetterer, 2011), intestine (Granger et al., 2011), and skeletal muscle (Shepherd, 1983), buffering organ blood flow and capillary pressure in the face of changes in blood pressure (Johnson, 1980; Davis et al., 2011). The myogenic response and myogenic tone also appear to contribute to blood pressure regulation by amplifying vasoconstrictor-induced vasoconstriction (Meininger and Trzeciakowski, 1988, 1990). Myogenic tone offers a resting level of smooth muscle contractile activity such that resistance arteries and arterioles can both dilate and constrict around their resting diameters, maintaining cardiovascular homeostasis (Renkin, 1984; Davis et al., 2011).

### Steps Involved in the Myogenic Response Leading to Myogenic Tone

Two questions need to be answered to understand the mechanisms responsible for the myogenic response and myogenic tone: (1) How are changes in luminal pressure sensed by smooth muscle cells in the wall of resistance arteries and arterioles? and (2) How are changes in luminal pressure transduced into vascular smooth muscle contraction and maintained tone in these vessels?

## How is Pressure Sensed in Resistance Arteries and Arterioles?

In a resistance artery or arteriole, increased luminal pressure results in a radial force that increases tangential wall stress (tension) as described by the Law of Laplace: tangential wall stress = *Pr*/Δ, where *P* is the pressure in the lumen of the vessel, *r* is the lumen radius, and Δ is the thickness of the vessel wall. The increased wall stress passively dilates the vessel and “stretches” (induces strain in) the smooth muscle cells in the vessel wall. The passive dilation will continue until either, the passive wall tension (due to collagen, elastin, cytoskeletal elements, etc.) matches the pressure-induced tangential wall stress, or the VSMCs actively contract and generate enough active wall stress to overcome the pressure-driven tangential wall stress.

**Figure 2:**
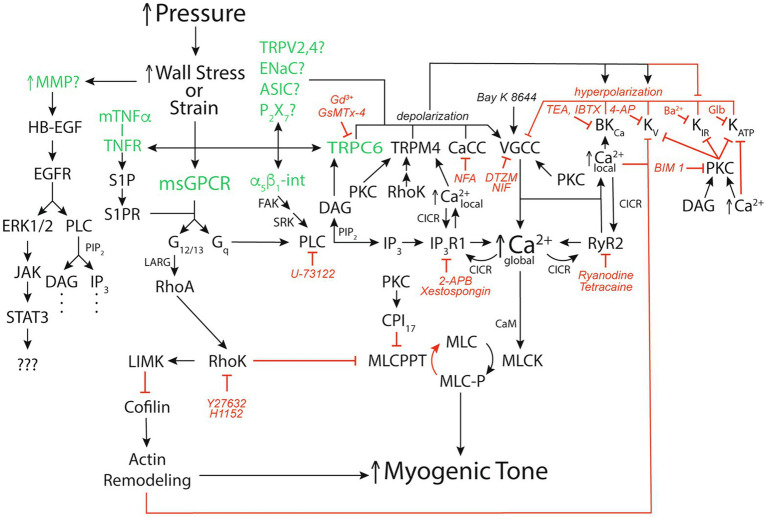
Signaling pathways for pressure-induced myogenic tone. Schematic diagram of reported signaling pathways involved in myogenic tone in resistance arteries and arterioles. Green font color depicts putative mechanosensors in pressure-induced myogenic tone. Black arrows show stimulation, increases or activation of signaling molecules, ion channels, or enzymes that participate in myogenic tone. Red capped lines indicate inhibition, decreases or deactivation of signaling molecules, ion channels, or enzymes involved in myogenic tone. Also shown are pharmacological agents that we have used to interrogate the ion channels and signaling pathways in arteriolar myogenic tone. MMP, matrix metalloproteinase; HB-EGF, heparin-bound epidermal growth factor; EGFR, epidermal growth factor receptor; ERK1/2, extracellular-signal-related kinases 1 or 2; JAK, janus kinase; STAT3, signal transducer and activator of transcription 3; mTNFα, membrane-bound tumor necrosis factor α; TNFR, TNFα Receptor; S1P, sphingosine-1-phosphate; S1PR, S1P receptor; α_5_β_1_-int, α_5_β_1_ integrin: FAK, focal adhesion kinase; SRK, Src-related kinases; CaCC, Ca^2+^-activated Cl^−^ channel; TRPV2,4, transient receptor potential vanilloid-family 2 or 4 channels; ENaC, epithelial Na^+^ channel; ASIC, acid sensing ion channel; P_2_X_7_, P_2_X_7_ purinergic receptor; VGCC, voltage-gated Ca^2+^ channel; BK_Ca_, large-conductance Ca^2+^-activated K^+^ channel; K_V_, voltage-gated K^+^ channel; K_IR_, inwardly-rectifying K^+^ channel; K_ATP_, ATP-sensitive K^+^ channel; msGPCR, mechanosensitive G-protein-coupled receptor; DAG, diacylglycerol; PKC, protein kinase C; NFA, niflumic acid; DTZM, diltiazem; NIF, nifedipine; TEA, tetraethylammonium; IBTX, iberiotoxin; 4-AP, 4-aminopyridine; GLIB, glibenclamide; BIM I, bisindolylmaleimide I; PLC, phospholipase C; PIP_2_, phosphatidylinositol bisphosphate; IP_3_, inositol, 1,4,5 trisphosphate; IP_3_R1, IP_3_ receptor 1; RyR, ryanodine receptor; CICR, Ca^2+^-induced-Ca^2+^ release; LARG, guanine nucleotide exchange factor LARG; RhoA, small G-protein Rho; 2-APB, 2-Aminoethoxydiphenyl borate; RhoK. Rho kinase; LIMK, LIM kinase; CPI_17_, C-kinase potentiated protein phosphatase-1 Inhibitor; MLCPPT, myosin light-chain phosphatase; MCL, myosin light-chain; MLCK, myosin light-chain kinase; See text for more details and references.

It has been argued that wall strain (change in length) is unlikely to be the main variable sensed in the myogenic response because, for example, arterioles, often constrict to diameters below their starting point with step increases in pressure (Johnson, 1980; Carlson and Secomb, 2005). However, cytoskeletal remodeling appears to occur coincident and in parallel with, smooth muscle contraction and relaxation such that cell “length” is not a constant (Gunst and Zhang, 2008). Thus, whether it is stress or strain that is sensed in the vessel wall remains to be clarified (Hill and Meininger, 1994; Davis and Hill, 1999; Hill et al., 2001; Davis et al., 2011). Nonetheless, several mechanisms have been proposed to act as sensors during the myogenic response leading up to steady-state myogenic tone including: several G-protein coupled receptors (Brayden et al., 2013; Schleifenbaum et al., 2014; Storch et al., 2015; Kauffenstein et al., 2016; Mederos et al., 2016; Hong et al., 2017; Pires et al., 2017; Chennupati et al., 2019), several cation channels (Welsh et al., 2002; Jernigan and Drummond, 2005; Gannon et al., 2008; VanLandingham et al., 2009; Nemeth et al., 2020), integrins (Davis et al., 2001; Martinez-Lemus et al., 2005; Colinas et al., 2015), matrix metalloproteinases (MMPs), and epidermal growth factor receptors (EGFR; Lucchesi et al., 2004; Amin et al., 2011); and membrane-bound tumor necrosis factor α (mTNFα), TNFα receptor (TNFR), and downstream sphingosine-1-phosphate (S1P) signaling (Kroetsch et al., 2017; Figure 2). What remains unclear is what determines which of these putative mechanosensitive elements are expressed in a particular blood vessel and how this expression is controlled under different physiological and pathological conditions. It is also not clear whether the different potential mechanosensitive elements represent independent “sensors” or whether some are linked together. For example, while there is evidence that TRPC6 channels are mechanosensitive and could serve as independent sensors of pressure-induced stress/strain in vascular smooth muscle (Spassova et al., 2006), it is also quite clear that these channels lie downstream from mechanosensitive G-protein coupled receptors, like the angiotensin II type 1 receptors (AT1R) that appear to mediate myogenic reactivity in rodent cerebral resistance arteries (Gonzales et al., 2014). Another example is the potential link between G-protein coupled receptors, such as the AT1R and the EGFR. There is considerable evidence for transactivation of the EGFR and its downstream targets upon activation of AT1R by angiotensin II (Forrester et al., 2016). Thus, it seems likely that mechanical activation of AT1R would do the same and may reconcile studies identifying EGFR as a key component of myogenic signaling in coronary artery vascular smooth muscle (Lucchesi et al., 2004; Amin et al., 2011) with studies identifying AT1R as the key mechanosensor in myogenic tone (Mederos y Schnitzler et al., 2008; Yasuda et al., 2008; Storch et al., 2012; Gonzales et al., 2014; Mederos et al., 2016; Hong et al., 2017; Pires et al., 2017). However, it is not clear how signaling downstream from the EGFR involving extracellular signal-related kinases 1 and 2 (ERK1/2), janus kinase (JAK), and signal transducer and activator of transcription 3 (STAT3; Amin et al., 2011) fits into the overall scheme of myogenic tone. Similarly, while a role for mTNFα, its receptor and downstream S1P have been proposed to mediate myogenic tone (Peter et al., 2008; Lidington et al., 2009; Yang et al., 2012; Hui et al., 2015; Yagi et al., 2015; Sauve et al., 2016; Kroetsch et al., 2017), it is not clear how this mechanism “fits” with the bulk of data supporting membrane depolarization and activation of voltage-gated Ca^2+^ channels (VGCCs) as the fundamental basis for myogenic tone. Certainly, activation of PLCγ is a potential downstream signal in the S1P-pathway feeding into the same signaling pathway that has been proposed for AT1R, for example Gonzales et al. (2014). In addition, S1P signaling reportedly can lie downstream from the AT1R (Wilson et al., 2015). Unfortunately, few investigators have tried to perform critical tests of alternative hypotheses for all the proposed mechanisms underlying myogenic tone to try and sort out which mechanisms are functional in a particular blood vessel. In addition, most investigators tend to focus on single or at most a few blood vessels such that the generality of proposed mechanisms remains unclear and will require additional research. Investigators are encouraged to explore multiple mechanisms in arteries/arterioles from different vascular beds so that patterns can be better assessed.

Finally, it is not yet known exactly how membrane proteins are activated by increases in membrane stress or strain. Based on studies in other systems, membrane-bound proteins may be activated or altered by forces transmitted *via* connections with the extracellular matrix and/or the cytoskeleton, by changes in membrane curvature and/or by membrane thinning-induced protein conformational changes (Leiphart et al., 2019).

### How Are Changes in Luminal Pressure Transduced Into Myogenic Tone?

Regardless of the precipitating mechanisms, *in vitro*, pressure myography studies have repeatedly shown that pressure-induced myogenic tone involves vascular smooth muscle membrane depolarization, activation of VGCCs, and an increase in intracellular Ca^2+^ (Harder, 1984; Knot and Nelson, 1998; Kotecha and Hill, 2005; Figure 2). As will be discussed in a subsequent section, there also may be activation of the small G-protein RhoA and the Rho kinase pathway which induces Ca^2+^ sensitization (Lagaud et al., 2002; Moreno-Dominguez et al., 2013) and Rho-kinase (Moreno-Dominguez et al., 2013) or protein kinase C (PKC)-dependent remodeling of the actin cytoskeleton (Moreno-Dominguez et al., 2013; Hong et al., 2016) that also can contribute to myogenic tone (Figure 2).

### Which Ion Channels Contribute to Pressure-Induced Membrane Depolarization?

A number of ion channels have been proposed to contribute to pressure-induced depolarization of VSMCs (Harder, 1984; Knot and Nelson, 1998; Kotecha and Hill, 2005) including: members of the transient receptor potential (TRP)-family of cation channels, such as TRPC6 (Welsh et al., 2002; Gonzales et al., 2014), TRPM4 (Earley et al., 2004; Gonzales et al., 2014), TRPV2 (McGahon et al., 2016), and TRPV4 (Soni et al., 2017); members of the degenerin family of channels including the epithelial Na^+^ channel (ENaC) family (Jernigan and Drummond, 2005, 2006; VanLandingham et al., 2009; Drummond and Stec, 2015), the acid-sensitive ion channel (ASIC) family of channels (Gannon et al., 2015), and purinergic P_2_X_7_ Purinergic Receptor (P_2_X_7_) channels (Kauffenstein et al., 2016; Figure 2). In addition, Ca^2+^-activated Cl^−^ channels (CaCC; ANO1/TMEM16A) may be activated by Ca^2+^ influx through TRPC6 channels (Bulley et al., 2012; Wang et al., 2016) and also contribute to pressure-induced depolarization of VSMCs (Figure 2). Finally, the activity of several K^+^ channels may be inhibited by either the membrane depolarization [inwardly-rectifying K^+^ channel (K_IR_) channels], increases in intracellular Ca^2+^ [voltage-gated K^+^ channel (K_V_) and ATP-sensitive K^+^ channel (K_ATP_) channels], or increased activity of kinases such as protein kinase C (K_V_ and K_ATP_ channels) and Rho kinase (K_V_ channels) and indirectly contribute to pressure-induced depolarization (Tykocki et al., 2017; Figure 2). As for other aspects of myogenic tone, there appears to be vascular bed- and, perhaps, species-dependent differences in the ion channels that participate in pressure-induced depolarization making generalizations difficult. Our understanding of the mechanisms responsible for this heterogeneity is lacking.

### What Activates the Ion Channels Responsible for Pressure-Induced Depolarization?

The answer to this question remains incomplete. In cerebral resistance arteries, TRPC6 and TRPM4 appear to be activated indirectly (Gonzales et al., 2014), likely downstream from pressure-induced activation of AT1R and phospholipase Cγ (PLCγ; Gonzales et al., 2014) with diacylglycerol (DAG) from hydrolysis of membrane phosphatidyl-inositol-bis-phosphate (PIP_2_) activating TRPC6 and Ca^2+^ from Ca^2+^- and IP_3_-induced release of Ca^2+^ through IP_3_ receptor (IP_3_R) activating TRPM4 (Gonzales et al., 2014; Figure 2). In cerebral penetrating arterioles, Rho kinase may be involved in the activation of TRPM4 by setting its Ca^2+^ sensitivity (Li and Brayden, 2017; Figure 2). However, not all blood vessels appear to require PLC for pressure-induced myogenic tone. For example, we have shown that myogenic tone and global Ca^2+^ levels in hamster cheek pouch arterioles are unaffected by inhibition of PLC and IP_3_R, and yet are inhibited by nanomolar concentrations of Gd^3+^ and micromolar concentrations of GsMTX-4, established inhibitors of mechanosensitive ion channels (Jackson and Boerman, 2017). Thus, it is possible that, in cheek pouch arterioles, pressure-induced mechanical activation of TRPC6, for example, directly contributes to membrane depolarization and myogenic tone (Figure 2).

### Which Ion Channels Contribute to the Negative-Feedback Regulation of Myogenic Tone?

Membrane depolarization in cells that express VGCCs is inherently a positive feedback process that would lead to depolarization approaching the Nernst equilibrium potential for Ca^2+^ (approximately +60 mV) and maximal vasoconstriction if it were not for negative feedback mechanisms that limit membrane depolarization and the activity of VGCCs. In VSMCs, this negative feedback is provided by large-conductance Ca^2+^-activated K^+^ (BK_Ca_) channels and several voltage-gated K^+^ (K_V_) channel family members including K_V_1.5, 2.1, and 7.X channels (Tykocki et al., 2017; Figure 2). The membrane depolarization induced by TRPC6, TRPM4, etc. activates both BK_Ca_ and K_V_ channels, limiting membrane depolarization (Tykocki et al., 2017; Figure 2). The BK_Ca_ channels are also activated by increased Ca^2+^ (Tykocki et al., 2017). In resistance arteries, the source of Ca^2+^ responsible for activation of BK_Ca_ channels is Ca^2+^ sparks released through ryanodine receptors (RyR; Nelson et al., 1995; Westcott and Jackson, 2011; Westcott et al., 2012), whereas in downstream arterioles, BK_Ca_ channels appear to be activated by Ca^2+^ entry through VGCCs and other membrane channels (Guia et al., 1999; Westcott and Jackson, 2011; Westcott et al., 2012; Suzuki et al., 2013; Figure 2).

### Which Ion Channels Contribute to Pressure-Induced Increases in Global Intracellular Ca^2+^?

L-type VGCCs composed of CaV1.2 α-pore-forming subunits contribute substantially to pressure-induced myogenic tone that is observed in pressurized resistance arteries and arterioles studied *ex vivo* (Tykocki et al., 2017; Figure 2). L-type CaV1.2 VGCCs appear essential for the initiation of the myogenic response because block of these channels prevents the development of myogenic tone (Knot and Nelson, 1998; Kotecha and Hill, 2005). In rat middle cerebral arteries, the voltage dependence of intracellular Ca^2+^ and myogenic tone is the same as that for currents through L-type VGCCs, and both depolarization-induced increases in intracellular Ca^2+^ and myogenic tone are prevented or completely reversed by L-type VGCC blockers (Knot and Nelson, 1998). In first-order rat cremaster muscle arterioles, the relationship between membrane potential and tone is steeper than observed in cerebral arteries, and a significant portion of pressure-induced tone remains after block of L-type VGCCs (Kotecha and Hill, 2005). Block of L-type VGCCs also only inhibits a portion of Ca^2+^-dependent myogenic tone in second-order hamster cremaster (86%) and cheek pouch (54%) arterioles (Jackson and Boerman, 2017). These data suggest that Ca^2+^ entry through additional ion channels, such as T-type VGCCs (CaV3.X) or mechano-sensitive cation channels such as TRPC6, for example, also contribute to elevated [Ca^2+^]_in_ and activation of contraction, particularly in the microcirculation (Tykocki et al., 2017). While it has been shown that smooth muscle-specific knockout of CaV1.2 abolishes myogenic reactivity in murine tibialis arteries (Moosmang et al., 2003), these data are difficult to interpret because CaV1.2 appears essential for the initiation of the myogenic response and hence, myogenic tone (Knot and Nelson, 1998; Kotecha and Hill, 2005).

The role played by SMC CaV1.2 channels in myogenic tone in arterioles, *in situ*, is not as clear. While there are a number of *in situ* studies supporting a role for CaV1.2 channels in various vascular beds (see Tykocki et al., 2017 for refs.), several intravital microscopy studies of arterioles in rat (Hill and Meininger, 1994), hamster (Jackson, 2012) and mouse (Pemberton et al., 1996; Ngo et al., 2013) cremaster muscles, and hamster cheek pouch (Boric et al., 1990; Welsh et al., 1998) have shown that topical application of L-type channel blockers has little effect on resting myogenic tone. Importantly, the efficacy the Ca^2+^ channel blockers was verified because they abolished vasomotion (Hill and Meininger, 1994; Welsh et al., 1998; Jackson, 2012; Ngo et al., 2013) and prevented O_2_-induced vasoconstriction (Welsh et al., 1998; Jackson, 2012). The resting tone in these studies appeared to be voltage-dependent because K_ATP_ channel agonists such as pinacidil (Jackson, 1993; Hill and Meininger, 1994) and cromakalim (Jackson, 1993) cause near maximal dilation of arterioles in these preparations. These data suggest that some other voltage-dependent channel, such as T-type CaV3.X channels for example, can determine resting tone in these microvascular beds under the conditions studied, that is simply not recapitulated in the *ex vivo* study of isolated resistance arteries and arterioles where CaV1.2 channels appear essential.

In some resistance arteries and arterioles, Ca^2+^ influx through VGCCs also appears to be amplified by Ca^2+^ release from intracellular stores (Figure 2). In cremaster arterioles, for example, Ca^2+^ influx through L-type VGCCs activates Ca^2+^ release through IP_3_Rs in the form of Ca^2+^ waves that contribute to myogenic tone (Westcott and Jackson, 2011; Westcott et al., 2012; Jackson and Boerman, 2018). In resistance arteries upstream from cremaster arterioles, Ca^2+^ waves also contribute to myogenic tone, but appear to involve both IP_3_R and RyR (Westcott and Jackson, 2011; Westcott et al., 2012).

### How Is the Pressure-Dependent Ca^2+^ Signal Translated Into Tone?

The global increase in intracellular Ca^2+^ that results from activation of membrane mechanoreceptive processes, depolarization, activation of VGCCs, and amplification by Ca^2+^ release from intracellular stores is translated into smooth muscle contraction mainly through binding of Ca^2+^ to the Ca^2+^-binding protein, calmodulin (CaM), and Ca^2+^-CaM-dependent activation of myosin light-chain kinase (MLCK). This results in phosphorylation of the 20 kD myosin light-chains which is the primary trigger for contraction and force production in vascular smooth muscle (Zou et al., 1995; Cole and Welsh, 2011; Figure 2). Myosin light-chain phosphorylation then allows interaction of filamentous actin with myosin, the formation of actin-myosin cross-bridges, cross-bridge cycling, and smooth muscle contraction or force generation (increased myogenic tone; Zou et al., 1995; Cole and Welsh, 2011; Figure 2). This process continues while Ca^2+^ remains elevated and cross-bridge cycling occurs. A reduction in intracellular Ca^2+^ or dephosphorylation of the myosin light-chains by myosin light-chain phosphatase (MLCPPT) turns off this process and allows smooth muscle relaxation (decreased myogenic tone; Zou et al., 1995; Cole and Welsh, 2011). The ratio of activity of MLCK/MLCPPT determines the Ca^2+^ sensitivity of the system (Cole and Welsh, 2011). Guanine nucleotide exchange factors (GEFs), such as LARG, couple G-proteins, such as G_12/13_ to activation of the small GTPase, RhoA which subsequently activates Rho Kinase (Chennupati et al., 2019; Figure 2). Active Rho Kinase has several targets that modulate myogenic tone including: phosphorylation and inhibition of MLCPPT and an increase in Ca^2+^ sensitivity (Cole and Welsh, 2011); activation of LIM kinase (LIMK) and subsequent inhibition of cofilin and actin-cytoskeleton remodeling (Loirand et al., 2006; Moreno-Dominguez et al., 2013); inhibition of K_V_ channels (Luykenaar et al., 2009); activation of TRPM4 channels (Li and Brayden, 2017); and activation of VGCCs (Guan et al., 2019; Figure 2). All of these Rho Kinase-related effects promote increased myogenic tone. Myogenic tone can also be increased/sustained through G-protein-dependent activation of PKC that not only modulates ion channels, but also the Ca^2+^ sensitivity of the contractile machinery through phosphorylation of the protein CPI_17_ which inhibits MLCPPT (Cole and Welsh, 2011). As noted previously, PKC also can lead to actin cytoskeleton remodeling that contributes to myogenic tone (Moreno-Dominguez et al., 2013; Hong et al., 2016; Figure 2).

## What is Responsible for the Apparent Heterogeneity in Mechanisms Underlying Myogenic Tone?

As outlined in previous sections, there are likely multiple mechanisms that resistance arteries and arterioles use to produce and modulate myogenic tone depending on their location in the body and the physiology/pathophysiology of the system. What determines the primary mechanisms that are functional in a resistance artery or arteriole under a given set of physiological or pathophysiological conditions remains to be established. In experimental diabetes and subarachnoid hemorrhage, for example, it has been shown that there is apparent upregulation of the role played by mTNFα and S1P-signaling in myogenic tone of skeletal muscle (Sauve et al., 2016) and cerebral (Yagi et al., 2015) resistance arteries. However, the mechanisms responsible for this upregulation remain to be established. Another example is the differences that we have found in the mechanisms of myogenic tone in hamster cremaster vs. cheek pouch second-order arterioles (Jackson and Boerman, 2017). In cremaster arterioles, PLC and IP_3_R substantially contribute to Ca^2+^ signals (Ca^2+^ waves) and pressure-induced myogenic tone, whereas cheek pouch arterioles generate a similar level of tone that is independent of PLC and IP_3_R signaling (Jackson and Boerman, 2017). The mechanisms responsible for this regional heterogeneity are not known. Nonetheless, these differences in mechanisms likely mean that vascular smooth muscle in resistance arteries and arterioles has a “toolbox” of mechanisms that are potentially available to support the vital process of the myogenic response and myogenic tone in health and disease. Regional heterogeneity in mechanisms of myogenic tone may also provide new drug targets to treat vascular disease in an organ or tissue specific manner. For this to become a reality, much more research will be required to: (1) identify all of the potential signaling pathways that can contribute to myogenic tone in a selection of resistance arteries and arterioles from different vascular beds around the body using high density transcriptomic and proteomic approaches, understanding that many ion channels and receptors are normally expressed at very low levels in VSMCs, despite having major contributions to vessel function; (2) gain a better understanding of the regulation of message and protein expression for all these components using sophisticated pathway and informatic analysis of the “signals” detected in the transcriptomic and proteomic screens; and (3) perform appropriate functional *in situ* and *ex vivo* studies measuring diameter (as a readout of smooth muscle contraction at given levels of pressure), membrane potential, both local and global Ca^2+^ signals as well as careful biochemical assessment of pathway activity (protein phosphorylation, etc., see Moreno-Dominguez et al., 2013; for example) using conditional, cell-specific knockout, knockdown and knockin strategies as well as careful pharmacology to evaluate the role of the various signaling pathways that can contribute to myogenic tone in both resistance arteries and arterioles from around the body. This is a daunting task, but one that appears essential to move this field forward.

## Author Contributions

WJ conceived, wrote, and edited this manuscript and is solely responsible for its content. The content is solely the responsibility of the author and does not necessarily represent the official views of the National Institutes of Health.

### Conflict of Interest

The author declares that the research was conducted in the absence of any commercial or financial relationships that could be construed as a potential conflict of interest.
